# Blockade of Eosinophil-Induced Bronchial Epithelial-Mesenchymal Transition with a Geranyl Acetophenone in a Coculture Model

**DOI:** 10.3389/fphar.2017.00837

**Published:** 2017-11-16

**Authors:** Yu Z. Lee, Hui M. Yap, Khozirah Shaari, Chau L. Tham, Mohd R. Sulaiman, Daud A. Israf

**Affiliations:** ^1^Department of Biomedical Science, Faculty of Medicine and Health Sciences, Universiti Putra Malaysia, Seri Kembangan, Malaysia; ^2^Laboratory of Natural Products, Institute of Bioscience, Universiti Putra Malaysia, Seri Kembangan, Malaysia

**Keywords:** geranyl acetophenone, epithelial-mesenchymal transition, transforming growth factor beta, airway remodeling, asthma

## Abstract

Epithelial-mesenchymal transition (EMT) is currently recognized as the main cellular event that contributes to airway remodeling. Eosinophils can induce EMT in airway epithelial cells via increased transforming growth factor (TGF)-β production. We assessed the effect of synthetic 2,4,6-trihydroxy-3-geranyl acetophenone (tHGA) upon eosinophil-induced EMT in a cellular model. The human eosinophil cell line EoL-1 was used to induce EMT in BEAS-2B human bronchial epithelial cells. The induction of EMT was dose-dependently suppressed following tHGA treatment in which the epithelial morphology and E-cadherin expression were not altered. Protein and mRNA expression of vimentin, collagen I and fibronectin in eosinophil-induced epithelial cells were also significantly suppressed by tHGA treatment. Following pathway analysis, we showed that tHGA suppressed eosinophil-induced activator protein-1-mediated TGF-β production by targeting c-Jun N-terminal kinase and phosphoinositide 3-kinase signaling pathways. These findings corroborated previous findings on the ability of tHGA to inhibit experimental murine airway remodeling.

## Introduction

Airway remodeling is a major morbidity factor in asthma. It includes the disruption of epithelial integrity, subepithelial fibrosis, collagen deposition, smooth muscle cell hyperplasia, and hypertrophy, increased mucus production and submucosal gland and airway wall thickening (Barnes, 2008; Bergeron et al., 2010). Previously it was widely accepted that bronchospasm and inflammation surrounding the airways played a central role in the pathophysiology of asthma. However, in the last three decades, evidence suggests that defects and alteration of the bronchial epithelium greatly contribute to the airway structural remodeling that is highly associated with the manifestation of asthma (Al-Muhsen et al., 2011). Epithelial-mesenchymal transition (EMT) is currently recognized as a source for migrating mesenchymal cells (myofibroblasts and fibroblasts) that promote subepithelial fibrosis and extracellular matrix (ECM) deposition (Hackett, 2012; Pain et al., 2014). Transforming growth factor (TGF)-β is the most potent and well investigated EMT inducer in asthma (Ijaz et al., 2014). Yasukawa et al. (2013) reported recently that eosinophils can induce EMT in airway epithelial cells via increasing TGF-β production.

Studies have suggested corticosteroids, the gold standard in asthma treatment, as being ineffective in reversing airway structural changes (Doerner and Zuraw, 2009; Royce and Tang, 2009). Furthermore, currently there are no targeted therapies for reversing airway structural changes in asthma (Pascual and Peters, 2005). The geranyl acetophenone, 2,4,6-trihydroxy-3-geranyl acetophenone (tHGA), originally discovered in *Melicope ptelefolia*, contains a bioactive principle of the phloroglucinol core (Shaari et al., 2006). We previously demonstrated that synthetic tHGA inhibited airway inflammation in ovalbumin (OVA)-induced murine experimental asthma (Ismail et al., 2012). Recently, we also demonstrated that tHGA is orally active and able to exert a dose-dependent inhibitory effect on airway remodeling in a similar model (Lee et al., 2017).

In this communication, we demonstrate that tHGA can inhibit eosinophil-induced EMT by suppressing epithelial TGF-β1 production in an *in vitro* co-culture system of human eosinophils and bronchial epithelial cells. Inhibition was attained through modulation of c-Jun N-terminal kinase (JNK) and phosphoinositide 3-kinase (PI3K)/ protein kinase B (AKT) pathways. Our findings provide further insights into the molecular pathophysiological events altered by tHGA and support its investigation as a new non-steroidal oral lead for the management of allergic asthma.

## Materials and methods

### tHGA synthesis

A previous communication explains the synthesis and structure of tHGA (Lee et al., 2017). Briefly, a well-stirred mixture of phloracetophenone (1.000 g, 6 mmol), geranyl bromide (0.876 g, 4.80 mmol), and anhydrous potassium carbonate (0.415 g, 3.00 mmol) in dry acetone (3.5 ml) was refluxed for 6 h. The reaction mixture was filtered and evaporated under reduced pressure to give an oily orange residue that was purified by flash column chromatography on Si gel (petroleum ether-ethyl acetate, 10:1) to afford tHGA as a light-yellow powder; m.p. 128–130°C. Purity was more than 99%.

### Cell culture

The human bronchial epithelial cell line, BEAS-2B was purchased from American Type Culture Collection (ATCC, USA). The human eosinophilic leukemia cell line, EoL-1 was purchased from RIKEN BioResource Center (RIKEN, Japan). BEAS-2B cells were grown at 37°C, 5% CO_2_, in DMEM supplemented with 6 mM L-glutamine, 10% FBS and 100 U/ml streptomycin and penicillin. BEAS-2B cells were subcultured at 80% confluency to avoid squamous epithelial differentiation. EoL-1 cells were grown at the same atmospheric conditions but in RPMI-1640 medium supplemented with 2 mM L-glutamine, 10% FBS and 100 U/ml streptomycin and penicillin. Cells with passage number below seven were used in all experiments.

### Co-culture experiments and tHGA treatment

The eosinophil-induced EMT model was adopted from Yasukawa et al. (2013). Prior to each experiment, BEAS-2B cells were cultured to 60–70% confluency in 6-well plates and serum-starved with 1% FBS for 24 h. EoL-1 cells were subjected to differentiation and maturation in media supplemented with 0.5 mM sodium n-butyrate (BA) at 5 × 10^5^ cells/ml for 5 days. Non-cytotoxic doses of tHGA were determined prior to further experiments by the MTT viability assay following 24 h incubation of cells with varying doses of tHGA. For the co-culture experiments, serum-starved BEAS-2B cells were pretreated with serially-diluted tHGA (30 to 7.5 μM) in 0.1% DMSO for 1 h. Following removal of tHGA treatment, cells were washed with sterile PBS. A total of 2 × 10^6^ cells/well of BA-differentiated EoL-1 in RPMI-1640 media were added to BEAS-2B cultures for 48 h. Subsequently, EoL-1 cells were removed from BEAS-2B cells by gentle pipetting and washed thrice with PBS prior to assay. Co-culture experimental groups were as follow:

1. Normal group (N): BEAS-2B cells
2. Control group (C): BEAS-2B cells coculture with EoL-1
3. 30 μM tHGA treated group (30): tHGA-pretreated BEAS-2B cells coculture with EoL-1
4. 15 μM tHGA treated group (15): tHGA-pretreated BEAS-2B cells coculture with EoL-1
5. 7.5 μM tHGA treated group (7.5): tHGA-pretreated BEAS-2B cells coculture with EoL-1

For TGF-β-induced EMT experiments, BEAS-2B were induced by adding 5 ng/ml recombinant TGF-β1 (Merck Millipore, USA) for 48 h. Experimental groups for TGF-β-induced experiments were as follow:

1. Normal group (N): BEAS-2B cells
2. Control group (C): BEAS-2B cells induced with TGF-β
3. tHGA treated group (30): 30 μM tHGA-pretreated BEAS-2B cells induced with TGF-β
4. tHGA treated group (15): 15 μM tHGA-pretreated BEAS-2B cells induced with TGF-β
5. tHGA treated. group (7.5): 7.5 μM tHGA-pretreated BEAS-2B cells induced with TGF-β
6. Drug control group (SB): 10 μM SB431542-pretreated BEAS-2B induced with TGF-β

In tHGA target identification experiments, serum-starved BEAS-2B cells were pretreated with 30 μM tHGA or respective internal control inhibitors (Table 1) in 0.1% DMSO for 1 h. Treatments were removed and cells were washed with PBS prior to co-culture of BA-differentiated EoL-1 with BEAS-2B for 1 h. EoL-1 cells were removed from BEAS-2B cells by gentle pipetting and washed thrice with PBS prior to assay. Experimental groups for tHGA target identification experiments were as follow:

1. Normal group (N): BEAS-2B cells
2. Control group (C): BEAS-2B cells coculture with EoL-1
3. tHGA treated group (30): 30 μM tHGA-pretreated BEAS-2B cells coculture with EoL-1
4. Internal control group (SP/ PD/ SB/ TCN/ LY): Respective inhibitor-pretreated BEAS-2B cells coculture with EoL-1

**Table 1:** List of internal control inhibitors for target identification experiments.

**Experimental Target**	**Inhibitor**	**Treatment Concentration**
JNK/c-Jun	SP600125 (SP)	10 μM
ERK1/2	PD98059 (PD)	25 μM
p38 MAPK	SB202190 (SB)	10 μM
AKT/GSK-3β	Triciribine (TCN)	10 μM
PI3K	LY294002 (LY)	10 μM

### Morphological analysis

BEAS-2B cells were photographed at 400x magnification. Images of three arbitrary fields were taken for each treatment group. Quantitative methods of morphological changes in EMT were adopted from Ren et al. (2015). Briefly, the centroid of a cell is determined. The radius ratio was obtained by dividing the maximum radius with the minimum radius of a cell measured from the centroid to the cell membrane. All measurements were taken with a Leica Microsystems microscope imaging software (Leica Microsystems, Germany). All whole cells in each field were measured.

### Western blot

Whole cell extracts of BEAS-2B cells were obtained with Chemicon Total Protein Extraction Kit (Merck Millipore, USA) whereas protein samples of cytoplasmic and nuclear fractions were extracted with NucBuster Protein Extraction Kit (Merck Millipore, USA) according to the manufacturer's instructions. Protein samples of 20 μg were electrophoresed on 8–12% SDS-polyacrylamide gels and transferred to 0.2 μm polyvinylidene fluoride membranes using a wet transfer system at 0.35 mA for 1.5 h (Bio-Rad Laboratories, USA). Membranes were blocked with 5% bovine serum albumin in Tris-buffered saline-tween 20 (TBS-T) for 1 h prior to overnight incubation at 4°C with respective antibodies (Table 2) in 5% BSA TBS-T. Following washing with TBS-T, membranes were hybridized with horse radish peroxidase (HRP)-conjugated goat anti-rabbit secondary antibody (1:5000; Santa Cruz Biotechnology, USA) or HRP-conjugated goat anti-mouse secondary antibody (1:5000; Santa Cruz Biotechnology, USA) for 1 h at room temperature. Membranes were then incubated with chemiluminescence reagent (Thermo Scientific, USA) for 1 min and imaged in a gel documentation system (Vilber Lourmat, Germany). Membranes were stripped and reprobed as required. Band intensities were quantified by ImageJ Image Processing Software (NIH, USA) and normalized by comparison to β-actin or Lamin A/C.

**Table 2:** List of antibodies and dilution used in western blots.

**Antibodies**	**Source**	**Dilution**
Rabbit monoclonal anti-E-cadherin	Cell Signaling Technology, USA	1:1000
Rabbit polyclonal anti-tenascin-C	Santa Cruz Biotechnology, USA	1:200
Rabbit polyclonal anti-vimentin	Santa Cruz Biotechnology, USA	1:200
Rabbit polyclonal anti-collagen I	Abcam, USA	1:5000
Rabbit monoclonal anti-fibronectin	Abcam, USA	1:400
Rabbit polyclonal anti-p-JNK	Cell Signaling Technology, USA	1:1000
Rabbit polyclonal anti-JNK	Cell Signaling Technology, USA	1:1000
Rabbit polyclonal anti-p-ERK1/2	Cell Signaling Technology, USA	1:1000
Rabbit polyclonal anti-ERK1/2	Cell Signaling Technology, USA	1:1000
Rabbit polyclonal anti-p-p38	Cell Signaling Technology, USA	1:1000
Rabbit polyclonal anti-p38	Cell Signaling Technology, USA	1:1000
Rabbit polyclonal anti-AKT	Cell Signaling Technology, USA	1:1000
Rabbit polyclonal anti-p-AKT(Ser473)	Cell Signaling Technology, USA	1:1000
Rabbit polyclonal anti-p-GSK-3β(Ser9)	Cell Signaling Technology, USA	1:1000
Rabbit polyclonal anti-p-c-Jun	Cell Signaling Technology, USA	1:1000
Rabbit monoclonal anti-AKT(Thr308)	Cell Signaling Technology, USA	1:1000
Rabbit monoclonal anti-GSK-3β	Cell Signaling Technology, USA	1:1000
Rabbit monoclonal anti-c-Jun	Cell Signaling Technology, USA	1:1000
Mouse monoclonal anti-β-actin	Santa Cruz Biotechnology, USA	1:5000
Mouse monoclonal anti-Lamin A/C	Santa Cruz Biotechnology, USA	1:5000

### RT-PCR

Total RNA was isolated and purified from BEAS-2B cells following co-culture with EoL-1 for 48 h using Qiagen RNeasy Plus Mini Kit (Qiagen, USA) according to the manufacturer's protocol. RNA concentration and purity were determined by using the Implen Nanophotometer P300 (Implen, USA) while RNA integrity was examined by formaldehyde agarose gel electrophoresis. RNA samples of 100 ng were used with Qiagen One-Step reverse transcriptase-polymerase chain reaction (RT-PCR) kit (Qiagen, USA) according to the protocol recommended by the manufacturer. The master mix was performed in an Eppendorf thermal cycler (Eppendorf, Germany) for reverse transcription at 50°C for 30 min, initial PCR activation at 95°C for 15 min, denaturation at 94°C for 30 s, annealing at 57°C for E-cadherin, collagen I, fibronectin and β-actin for 30 s, 59°C for vimentin and β-actin for 30 s, and 65°C for TGF-β and β-actin for 30 s, elongation at 72°C for 1 min and final extension at 72°C for 10 min. The sequences of the primers (IDT, USA) are listed in Table 3. PCR was carried out for 35 cycles. The reaction products from PCR were examined by 1.8% agarose gel electrophoresis containing 0.01% of ethidium bromide. PCR products in each gel electrophoresis were ran in parallel to a Low Molecular Weight DNA Ladder (NEB, USA). Band intensities were quantified by ImageJ Image Processing Software (NIH, USA) and normalized by comparison to the RT-PCR product of β-actin mRNA.

**Table 3:** List of primers used in RT-PCR.

**Primer**	**Sequence**	**Product Size (bp)**
E-cadherin	forward 5′-GGCCTGAAGTGACTCGTAACG-3′ reverse 5′-CAGTATCAGCCGCTTTCAGATTT-3′	126
Vimentin	forward 5′-GAGAACTTTGCCGTTGAAGC-3′ reverse 5′-GCTTCCTGTAGGTGGCAATC-3′	163
Collagen I	forward 5′-ATGTGGCCATCCAGCTGAC-3′ reverse 5′-GTTGGAGCCCTTGAGGAGC-3′	143
Fibronectin	forward 5′-CCACCCCCATAAGGCATAGG-3′ reverse 5′-GTAGGGGTCAAAGCACGAGTCATC-3′	341/434
TGF-β	forward 5′-GCCCTGGACACCAACTATTGC-3′ reverse 5′-GCTGCACTTGCAGGAGCGCAC-3′	336
β-actin	forward 5′-GCGTGATGGTGGGCATGG-3′ reverse 5′-GATGCCGTGCACGATGGG-3′	101

### TGF-β1 immunoassay

Spent media from co-culture experiments was collected and centrifuged to remove EoL-1 cells and debris. The concentration of secreted TGF-β1 was quantified using a commercial enzyme-linked immunosorbent assay kit (R&D Systems, USA) according to manufacturer's protocol and subjected to correction with media native TGF-β1 levels.

### AP-1 DNA binding assay

The binding activity of activator protein (AP)-1 in nuclear fractions was assayed using an electrophoretic mobility shift assay. AP-1 consensus double stranded oligonucleotide (5′-CGC TTG ATG ACT CAG CCG GAA-3′) (Santa Cruz Biotechnology, USA) was end-labeled with Pierce Biotin 3′ End DNA Labeling Kit (Thermo Fisher Scientific, USA). Briefly, 10 μg of nuclear protein samples were incubated with 100 fmol of oligonucleotides at room temperature for 20 min and separated on a 6% polyacrylamide gel. The specificity of binding was determined by 200-fold unlabeled oligonucleotide competition. The AP-1-DNA complexes were transferred onto 0.2 μm polyvinylidene fluoride membranes and incubated with chemiluminescence reagent (Thermo Scientific, USA) for 1 min and imaged in a gel documentation system (Vilber Lourmat, Germany). Band intensities were quantified by ImageJ Image Processing Software (NIH, USA).

### Statistical analysis

All experiments were performed a minimal of three times with triplicate samples. Data were analyzed using one-way analysis of variance. Significant differences between experimental groups were tested using Dunnett's *post hoc* test by comparing to induced-controls (Group C –eosinophils or TGF-β-induced BEAS-2B cells). All data is expressed as mean ± S.E.M. Differences are considered significant when *P* < 0.05.

## Results

### tHGA inhibits eosinophil-induced EMT

**Figure 1:**
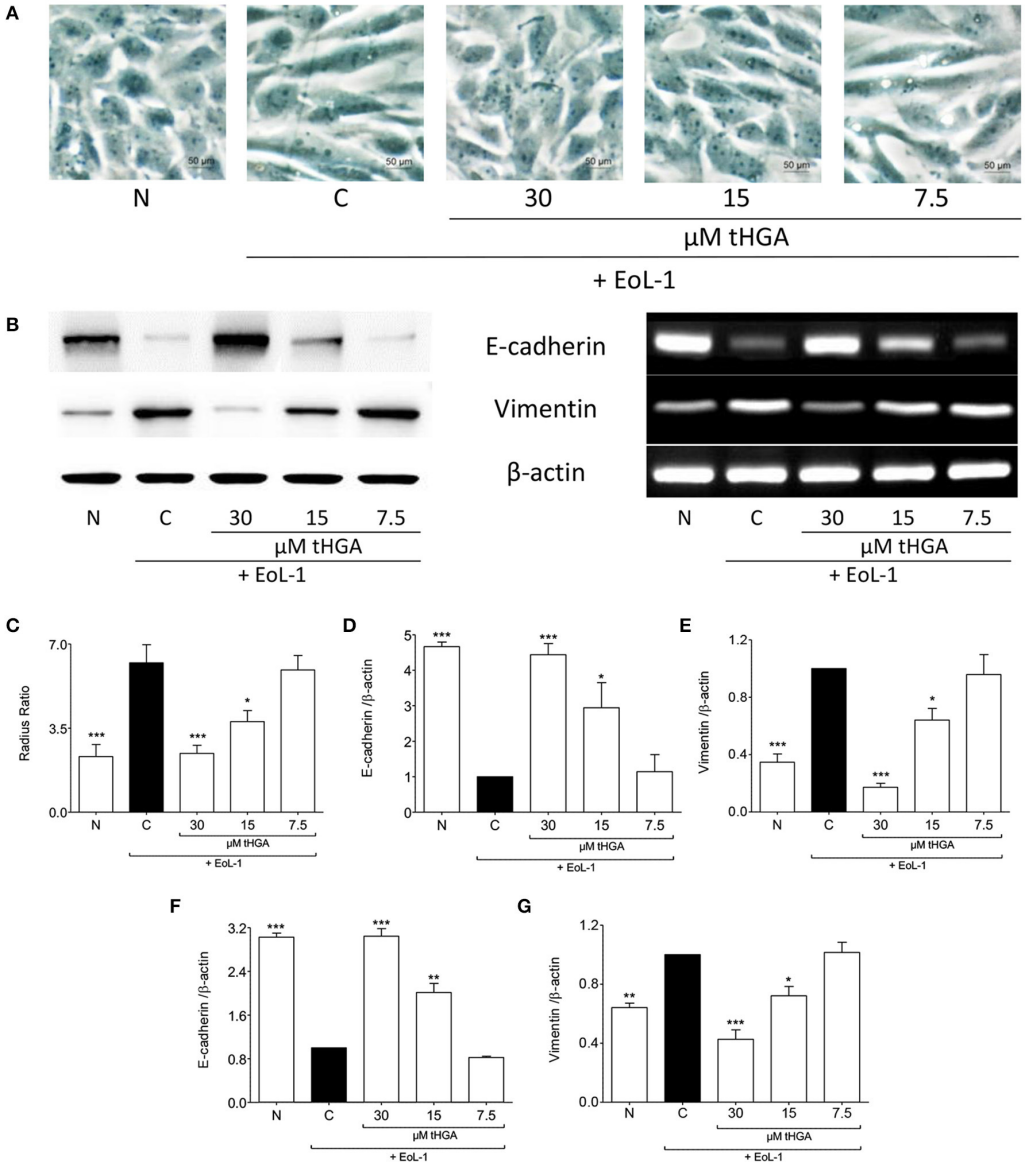
tHGA inhibits eosinophil-induced EMT. **(A)** Representative images of the morphology of normal BEAS-2B cells co-cultured with EoL-1 for 48 h in the presence or absence of tHGA pretreatment under x400 magnification. Bar = 50 μm. Quantitative data as measured by radius ratio in **(C)** were calculated by dividing the maximum radius with the minimum radius of a cell. **(B)** Protein expression (left panel) and mRNA expression (right panel) of E-cadherin and vimentin. Densitometric analysis with β-actin normalization of blots are presented in **(D,E)** and PCR gel images in **(F,G)**. All quantitative data are presented as mean ± S.E.M. of three independent experiments. Significant differences were compared to experimental group C: ^*^P < 0.05, ^**^P < 0.01, and ^***^P < 0.001. N, Normal control; C, Eosinophil-induced control.

As established previously (Yasukawa et al., 2013), the morphology of BEAS-2B cells was altered from cobble-stone shaped to spindle fibroblast-like morphology following co-culture with EoL-1, an indication of EMT (Figure 1A). Pretreatment with both 30 and 15 μM tHGA inhibited EMT as evident from the morphology and radius ratio of BEAS-2B cells (Figure 1C). Bronchial epithelial cells lose protein expression of the epithelial marker E-cadherin and start expressing the mesenchymal marker vimentin following co-culture with eosinophils. Treatment with tHGA prevented the loss of epithelial marker and mesenchymal marker upregulation from occurring (Figures 1B,D,E). Data further demonstrates that prevention of alterations in epithelial and mesenchymal marker expression were due to the effect of tHGA on gene expression (Figures 1F,G).

### tHGA prevents the expression of collagen I and fibronectin following eosinophil-induced EMT

**Figure 2:**
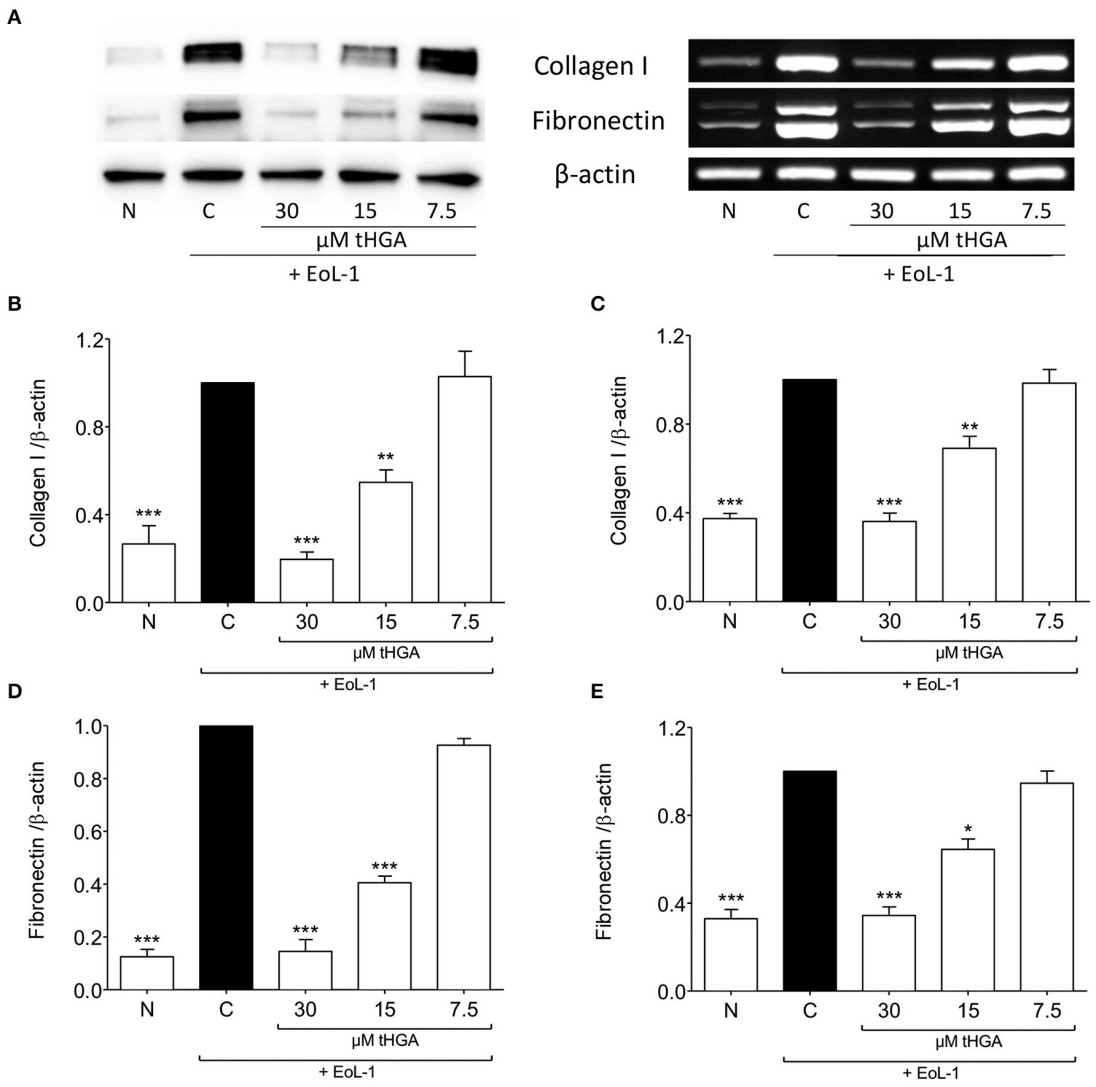
tHGA suppresses collagen I and fibronectin expression. **(A)** Protein expression (left panel) and mRNA expression (right panel) of collagen I and fibronectin of BEAS-2B cells prior to and after 48 h co-culture with EoL-1 in the presence or absence of tHGA pretreatment. Densitometric analysis with β-actin normalization of blots are shown in **(B)** for collagen I and **(D)** for fibronectin while PCR gel images in **(C)** for collagen I and **(E)** for fibronectin. All quantitative data are presented as mean ± S.E.M. of three independent experiments. Significance difference were compared to experimental group C: ^*^P < 0.05, ^**^P < 0.01 and ^***^P < 0.001. N, Normal control; C, Eosinophil-induced control.

The expression of fibronectin and collagen I which are uniquely associated with mesenchymal phenotype are shown in Figure 2. Western blots revealed that tHGA, particularly at a concentration of 30 μM, was effective in preventing fibronectin and collagen I protein expression which were significantly increased following EoL-1 co-culture. A similar trend was noted in the expression of the respective genes.

### tHGA suppresses eosinophil-induced Tgf-β expression by BEAS-2B

**Figure 3:**
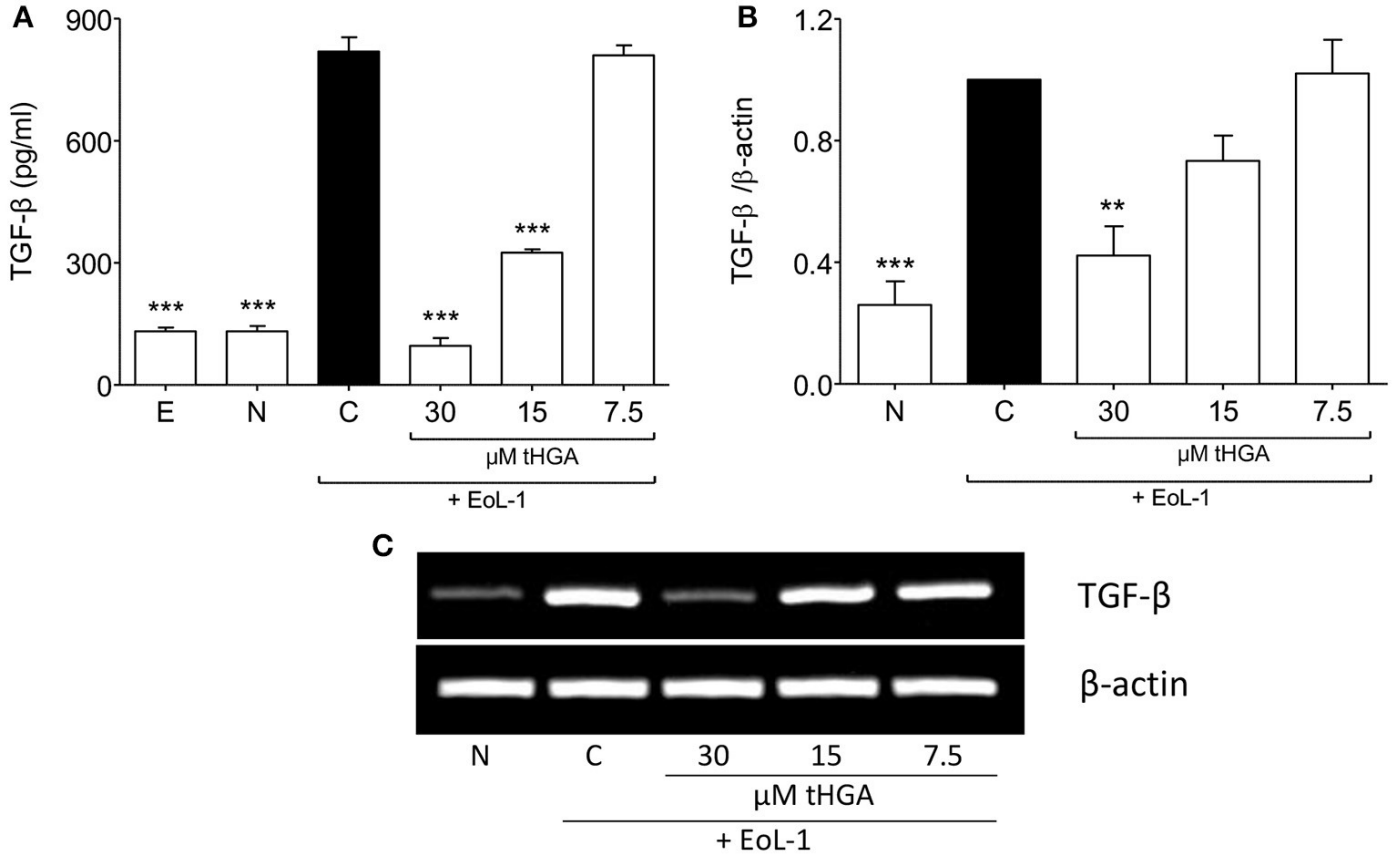
Suppression of eosinophil-induced TGF-β expression by tHGA. **(A)** Concentration of TGF-β1 in spent media was determined by enzyme-linked immunosorbent assay. **(B)** TGF-β1 mRNA expression in BEAS-2B as determined by densitometry analysis of RT-PCR product of TGF-β1 normalized with β-actin. PCR gel image is shown in **(C)**. All quantitative data are presented as mean ± S.E.M. of three independent experiments. Significance difference were compared to experimental group C: ^**^P < 0.01 and ^***^P < 0.001. E, EoL-1 only control; N, Normal BEAS-2B control; C, Eosinophil-induced control.

As described by Hosoki et al. (2014) and Yasukawa et al. (2013), TGF-β1 production was enhanced following co-culture of BEAS-2B with EoL-1. We demonstrated that that although monoculture of either cell line did not exhibit high TGF-β1 secretion, co-culture of BEAS-2B and EoL-1 significantly enhanced the release of TGF-β1 as determined by ELISA (Figure 3A). Interestingly, tHGA suppressed the secretion (Figure 3A) and mRNA expression (Figures 3B,C) of TGF-β1 by BEAS-2B co-cultured with EoL-1, in a concentration-dependent manner.

**Figure 4:**
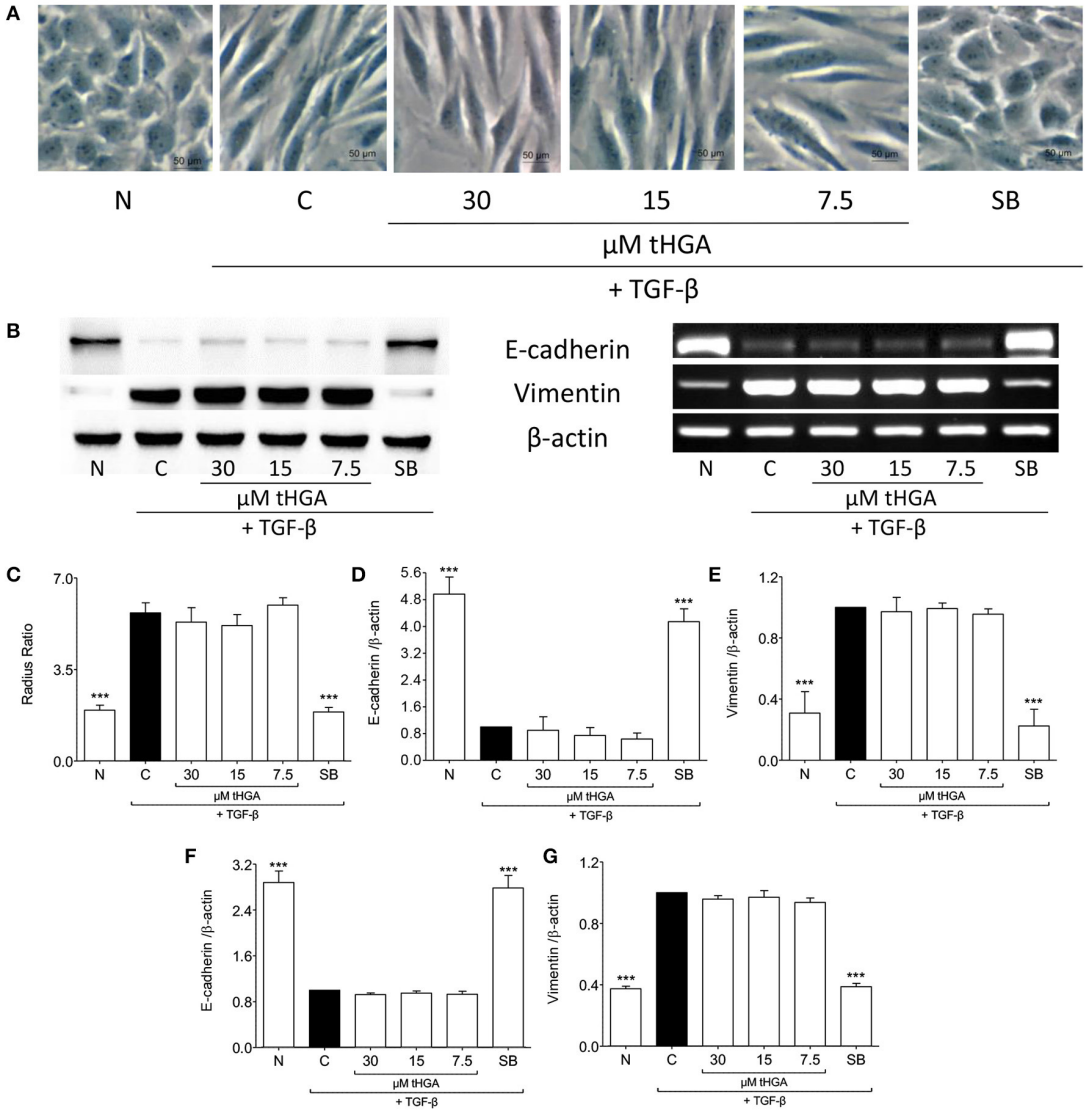
tHGA does not affect TGF-β-induced EMT. **(A)** Representative images of the morphology of normal BEAS-2B cells before and after induced with 5 ng/ml TGF-β for 48 h in the presence or absence of tHGA or 10 μM SB431542 pretreatment under x400 magnification. Bar = 50 μm. Quantitative data as measured by radius ratio is shown in **(C)** and calculated by dividing maximum radius with the minimum radius of a cell. **(B)** Protein expression (left panel) and mRNA expression (right panel) of E-cadherin and vimentin. Densitometric analysis with β-actin normalization of blots is presented in **(D)** for E-cadherin and **(E)** for vimentin while analysis for PCR gel images in **(F,G)**, respectively. All quantitative data are presented as mean ± S.E.M. of three independent experiments. Significance difference were compared to experimental group C: ^***^P < 0.001. N, Normal control; C, TGF-β-induced control; SB, 10 μM SB431542 treated control.

Since it has been suggested that EMT of eosinophil-induced bronchial epithelial cells is TGF-β dependent (Yasukawa et al., 2013), we assessed the effect of tHGA upon BEAS-2B cells induced by recombinant human TGF-β. Exposure of BEAS-2B cells to recombinant TGF-β induced altered morphology, loss of E-cadherin expression and increased vimentin expression. However, we observed no significant suppression of TGF-β-induced EMT following treatment with tHGA (Figure 4).

### tHGA blocks JNK but not ERK1/2 and p38 phosphorylation

**Figure 5:**
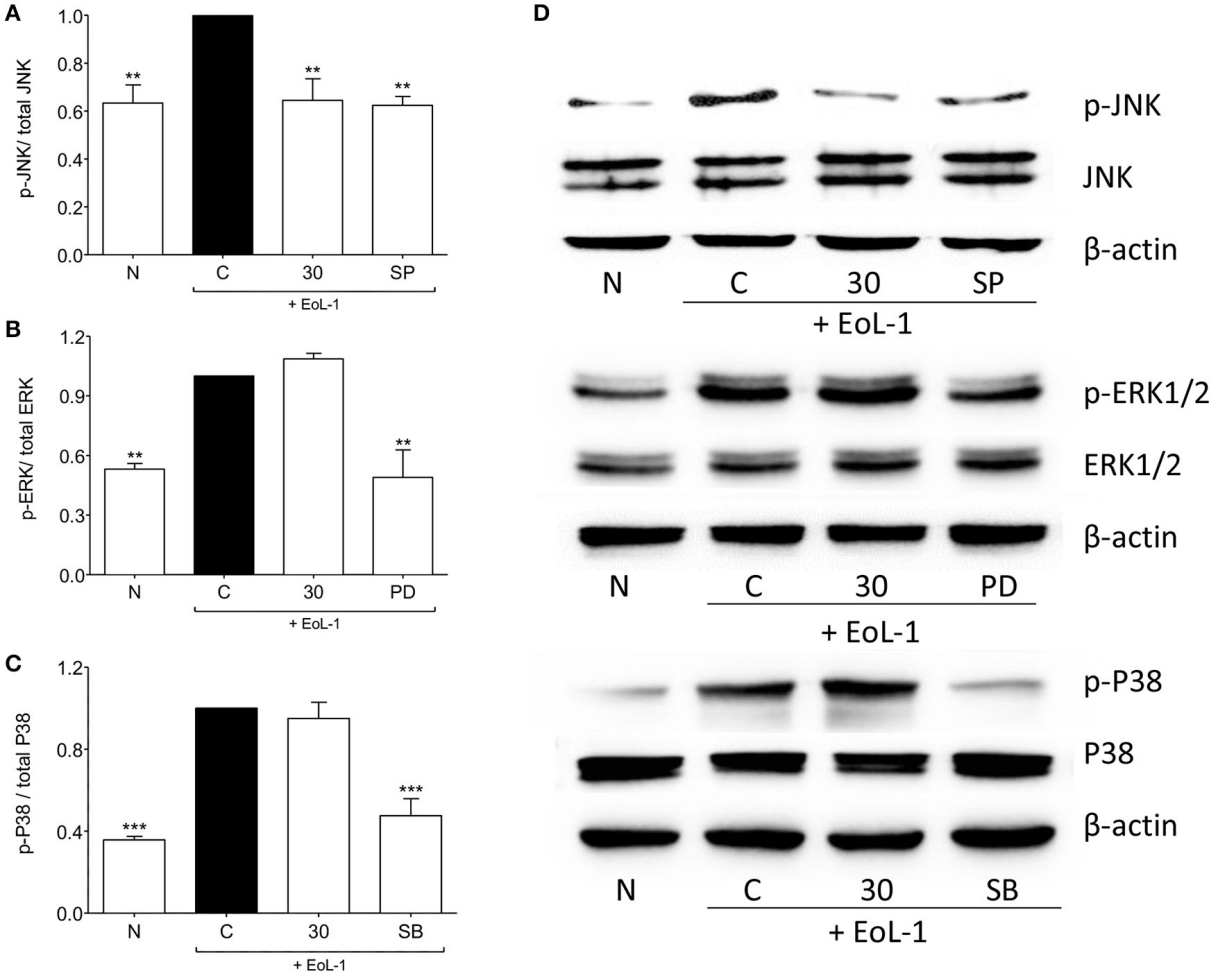
Effects of tHGA on MAPK pathway. Eosinophil-induced phosphorylation of JNK, ERK1/2, and p38 were assayed in whole cell extract of BEAS-2B in the presence or absence of tHGA or respective inhibitor pretreatment. Densitometry analysis of p-JNK, p-ERK1/2, and p-p38 normalized to total JNK, ERK1/2, and p38, respectively is presented in **(A–C)**, respectively. Representative blot for each parameter is shown in **(D)**. All quantitative data are presented as mean ± S.E.M. of three independent experiments. Significance difference were compared to experimental group C: ^**^P < 0.01 and ^***^P < 0.001. N, Normal control; C, Eosinophil-induced control; SP, 10 μM SP600125 treated control; PD, 25 μM PD98059 treated control; SB, 10 μM SB202190 treated control.

It was established previously that the activation of MAPK and PI3K pathways leads to the expression of TGF-β in BEAS-2B after co-culture with EoL-1 (Yasukawa et al., 2013). Figure 5 shows the effect of tHGA upon BEAS-2B cell MAPKs following EoL-1-induction. Induction successfully activated phosphorylation of all MAPKs. tHGA treatment caused significant inhibition of JNK phosphorylation (Figure 5A) without any effect upon ERK1/2 (Figure 5B) and p38 (Figure 5C).

### tHGA inhibits eosinophil-induced activation of the PI3K/ AKT/ GSK-3β

**Figure 6:**
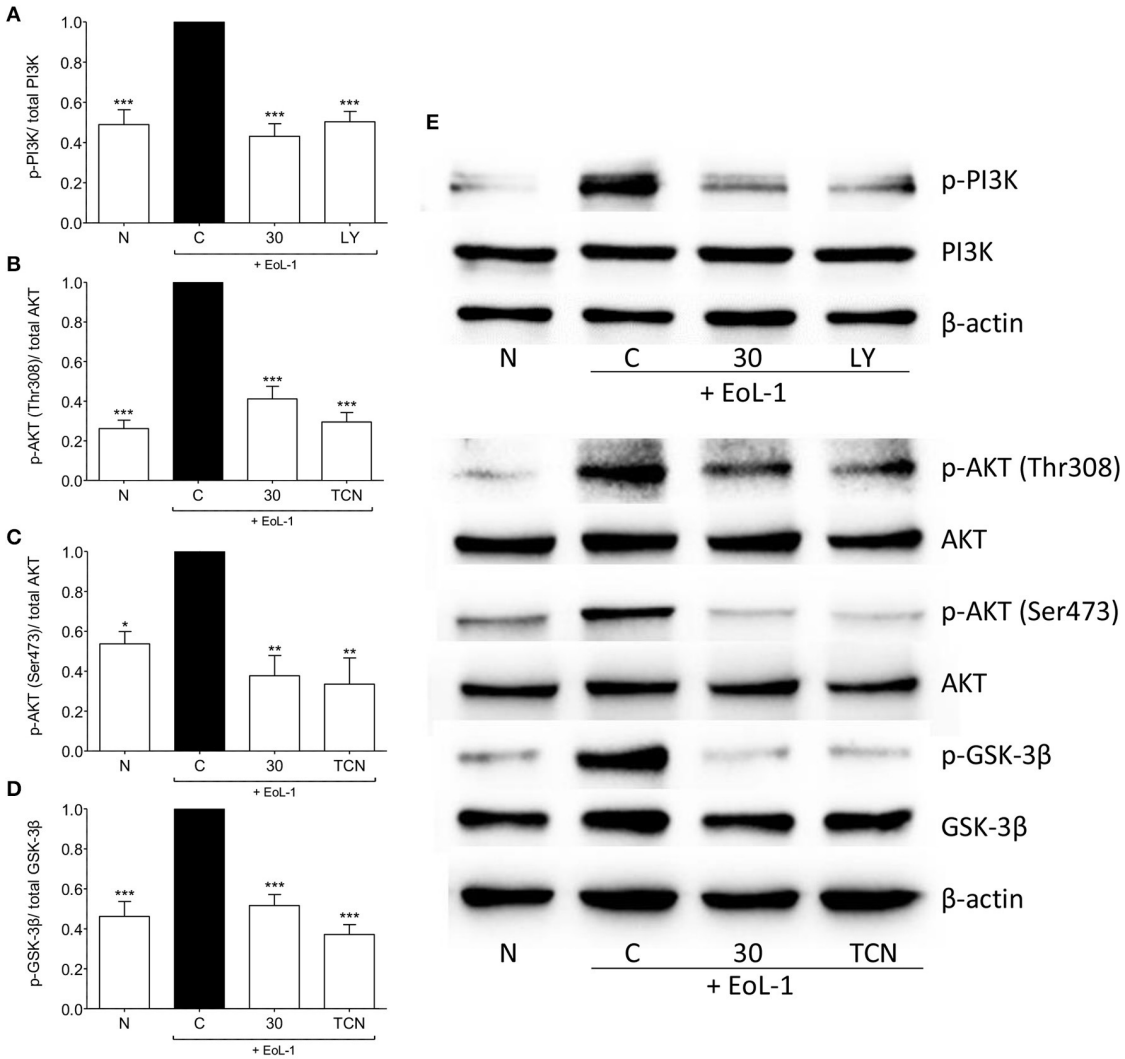
tHGA affects the activation of PI3K/ AKT/ GSK-3β pathway. Eosinophil-induced phosphorylation of PI3K, AKT, and GSK-3β were assayed in whole cell extract of BEAS-2B in the presence or absence of tHGA, LY294002 or triciribine pretreatment. Densitometry analysis of p-PI3K, p-AKT(Thr308), p-AKT(Ser473), and p-GSK-3β normalized to their respective total protein level is presented in **(A–D)**, respectively. Representative blot for each parameter is shown in **(E)**. All quantitative data are presented as mean ± S.E.M. of three independent experiments. Significance difference were compared to experimental group C: ^*^P < 0.05, ^**^P < 0.01, ^***^P < 0.001. N, Normal control; C, Eosinophil-induced control; LY, 10 μM LY294002 treated control; TCN, 10 μM triciribine treated control.

We examined the activation of the PI3K/AKT pathway and found that interaction between EoL-1 and BEAS-2B cells resulted in the phosphorylation of PI3K and AKT at both phosphorylation sites (Thr308 and Ser473) (Figures 6A–C,E). Activated AKT can phosphorylate GSK-3β at Ser9 which then inactivates GSK-3β (McCubrey et al., 2014). Since GSK-3β is associated with increased stability and availability of c-Jun for activation via phosphorylation (Wei et al., 2005), we also examined whether EoL-1/BEAS-2B co-culture leads to GSK-3β phosphorylation and whether this was blocked following tHGA treatment (Figures 6D,E). It was apparent that tHGA inhibited the phosphorylation of all these molecules.

### tHGA inhibits eosinophil-induced c-Jun activation and AP-1 DNA binding activity

**Figure 7:**
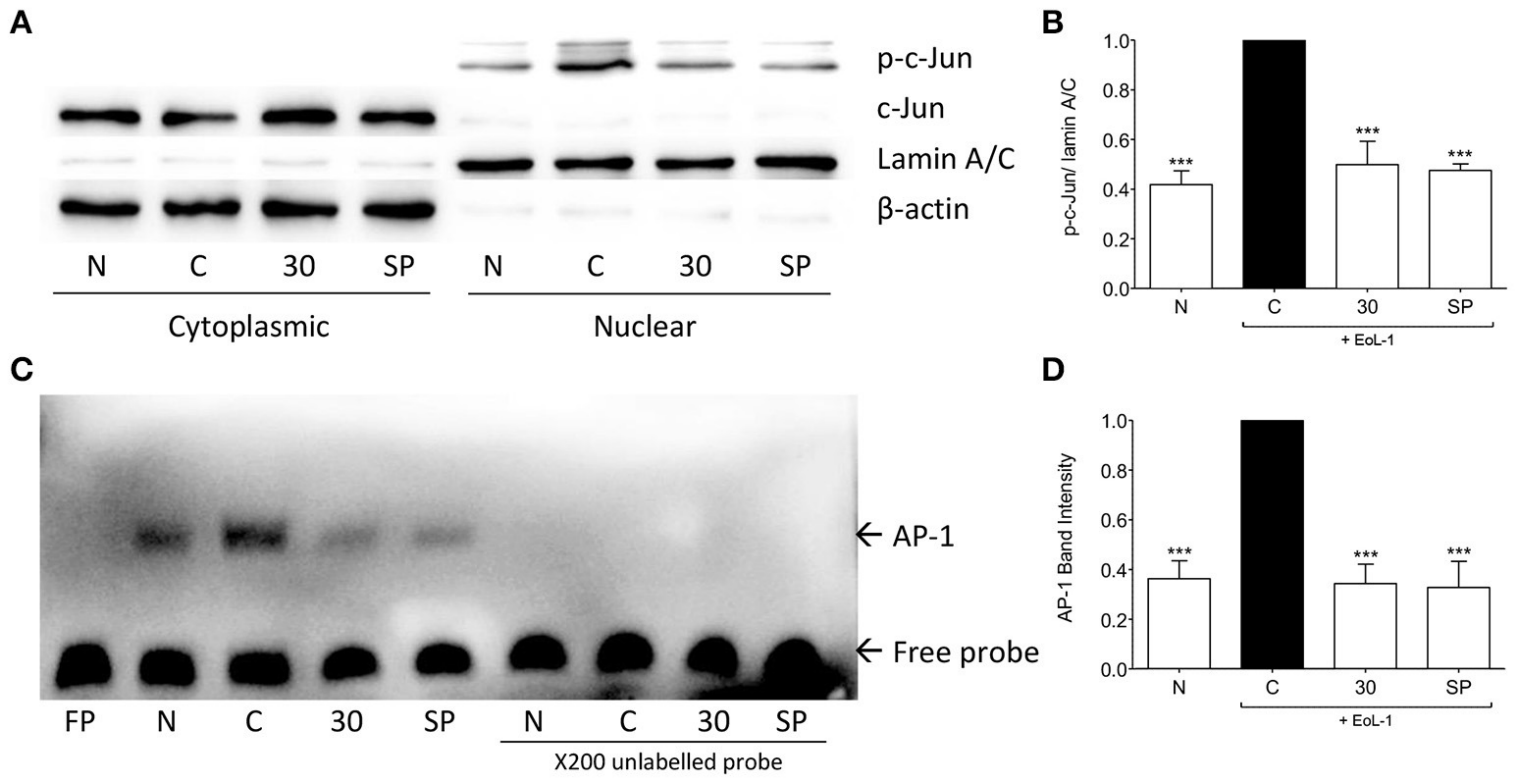
tHGA inhibits eosinophil-induced c-Jun activation and AP-1 DNA binding activity. BEAS-2B were pretreated with 30 μM tHGA or 10 μM SP600125 1 h prior to EoL-1 coculture for 1 h. **(A)** Cytosolic and nuclear fractions were subjected to western blot analysis for level of p-c-Jun. Densitometry analysis of p-c-Jun expression normalized to Lamin A/C in nuclear fraction is presented in **(B)**. Nuclear fractions were then assayed for DNA binding of AP-1 by electrophoretic mobility shift assay. Representative blot of AP-1 DNA binding is shown in **(C)** with mean densitometric data in **(D)**. All quantitative data are presented as mean ± S.E.M. of three independent experiments. Significance difference were compared to experimental group C: ^***^P < 0.001. N, Normal control; C: Eosinophil-induced control; SP: 10 μM SP600125 treated control.

AP-1 structurally consists of a homodimer of c-Jun or heterodimer of c-Jun and Fos or ATF protein families (Hess, 2004), it is a major regulator of TGF-β1 transcription (Sullivan et al., 2009). Following eosinophil induction c-Jun was phosphorylated and translocated into the nucleus of BEAS-2B cells. Treatment with tHGA inhibited c-Jun phosphorylation and translocation (Figures 7A,B). Since tHGA inhibited c-Jun phosphorylation we confirmed blockade of downstream AP-1 DNA binding through an electrophoretic mobility shift assay. As shown in Figures 7C,D, AP-1 DNA binding in BEAS-2B cells increased following EoL-1 co-culture whereas tHGA treatment caused a significant decrease in AP-1 DNA binding.

## Discussion

Recent work has provided substantial insights into the central role of airway epithelia in the pathogenesis of asthma (Al-Muhsen et al., 2011). EMT is currently recognized as the main cellular event that contributes to airway remodeling by being a source of myofibroblasts and fibroblasts (Hackett, 2012; Pain et al., 2014). Yasukawa et al. (2013) had reported recently that eosinophils can induce EMT in airway epithelial cells via increasing TGF-β production. By adopting their *in vitro* model, we have demonstrated prevention of EoL-1-induced BEAS-2B EMT by tHGA in which E-cadherin expression was not disrupted and vimentin not expressed. The increased expression of mesenchymal markers such as collagen I and fibronectin following eosinophil induction were correlated to the EMT process of primary airway epithelial cells (Hackett et al., 2009). The ability of tHGA to suppress the expression of collagen I and fibronectin further supports our previous observations of its inhibitory activity upon collagen and ECM deposition in sensitized mice (Lee et al., 2017).

TGF-β is the most potent and well investigated EMT inducer in asthma (Xu et al., 2009; Ijaz et al., 2014). Induction of EMT by eosinophils has also been demonstrated to be TGF-β dependent (Yasukawa et al., 2013). Recent evidence suggests that TGF-β is mainly derived from airway epithelial cells in contrast to previous suggestions that TGF-β is secreted by eosinophils during asthma pathogenesis (Makinde et al., 2007). Epithelial cells from asthma patient biopsies express TGF-β (Xu et al., 2003) and correlate well with basement membrane thickness and fibroblast numbers (Vignola et al., 1997). A study by Kumar et al. (2004) also showed that bronchial epithelia is the major source of TGF-β as opposed to eosinophils in a murine model of asthma. Although (Yasukawa et al., 2013) did not identify the source of TGF-β, their subsequent work demonstrated that montelukast pretreatment of BEAS-2B cells prior to co-culture suppressed EoL-1-induced TGF-β synthesis which strongly suggest that TGF-β is secreted by bronchial epithelial cells (Hosoki et al., 2014). We demonstrated significant increase in gene and protein expression of TGF-β in BEAS-2B cells following eosinophil induction. The increased gene expression and synthesis of TGF-β was suppressed by tHGA pretreatment of BEAS-2B cells. Thus, our findings further strengthen the suggestion that bronchial epithelial cells are the source of TGF-β in this particular co-culture model.

The increased synthesis of TGF-β in BEAS-2B cells following EoL-1 co-culture was suppressed by tHGA pretreatment and correlated with the attenuation of EMT. Although tHGA inhibited EoL-1-induced EMT, we demonstrated that this was not possible in a TGF-β-induced EMT model. TGF-β receptor activation mainly leads to the activation of the Smad signaling pathway that involves Smad2 or Smad3 associating with Smad4, forming a complex that regulates EMT-associated target gene expression (Makinde et al., 2007; Xu et al., 2009). The activation of the Smad signaling pathway alone, independent of the MAPK or PI3K pathway, has been shown to be sufficient in airway EMT induction (Hackett et al., 2009). Since tHGA inhibited eosinophil-induced EMT and TGF-β synthesis but not TGF-β-induced EMT, our findings implied that tHGA exerts its effect through the regulation of TGF-β production in epithelial cells instead of TGF-β-activated Smad signaling pathway.

Although the effect of TGF-β via its signaling pathway in EMT is well described, there is a lack of data pertaining to the transcriptional and translational regulation of TGF-β production in bronchial epithelial cells. Yasukawa et al. (2013) suggested the involvement of the JNK and PI3K pathways in EoL-1-induced EMT since treatment with their specific inhibitors abolished TGF-β production in the co-culture system. We demonstrated that EoL-1 induced the activation of MAPKs and PI3K/AKT pathways in BEAS-2B cells. In a study by Xiao et al. (2008), they showed that JNK, ERK1/2, and p38 activation enhanced TGF-β mRNA expression while TGF-β protein translation was regulated by the PI3K pathway. Our results show increases in both TGF-β mRNA and protein levels following eosinophil co-culture and tHGA successfully reduced both mRNA and protein expression of TGF-β. Treatment with tHGA significantly inhibited phosphorylation of JNK and PI3K without any effect upon phosphorylation of ERK1/2 and p38. These findings point to the possible pathway that is mediated by tHGA in suppression of TGF-β production.

Apart from translational regulation of TGF-β by the PI3K/AKT pathway, AKT may regulate c-Jun levels via GSK-3β. GSK-3β is natively active in phosphorylating c-Jun at Thr239, Ser243, and Ser249 which renders c-Jun inactive and subject to degradation (Boyle et al., 1991; Wei et al., 2005). Activated AKT can phosphorylate GSK-3β at Ser9 which then inactivates GSK-3β (McCubrey et al., 2014). Thus, c-Jun is stabilized and readily available for activation when GSK-3β is phosphorylated (Wei et al., 2005). Activation of JNK will lead to the phosphorylation of c-Jun and promote its transactivation potential through AP-1 formation (Karin et al., 1997). AP-1 is the most prominent transcription factor regulating TGF-β transcription as more than one AP-1 sites are found in two of the promoters with another three overlapping sites at about 200 base pairs downstream of the TGF-β gene (Birchenall-Roberts et al., 1990; Kim et al., 1990; Roberts et al., 1990; Scotto et al., 1990). It has been proven that AP-1 expression can induce EMT by modulating TGF-β expression in mammary epithelial cells (Bakiri et al., 2015). Since tHGA suppressed JNK and GSK-3β phosphorylation, we assessed c-Jun phosphorylation and AP-1 DNA binding activity and showed that tHGA significantly inhibited c-Jun phosphorylation and AP-1 activity, thus potentially affecting subsequent TGF-β transcription.

There are studies that demonstrate the critical role of ERK1/2 (Xie et al., 2004), PI3K (Bakin et al., 2000) and p38 (Yu, 2002) in TGF-β-induced EMT. However, the findings were derived from a mammary epithelial cell EMT model. Studies on airway epithelial cells revealed that p38 and ERK1/2 are non-essential in airway epithelial cell EMT as treatment of specific p38 and ERK1/2 inhibitors failed to fully inhibit TGF-β-induced EMT (Hackett et al., 2009; Câmara and Jarai, 2010). The expression of EMT markers and the extent of EMT progress were found to be cell type dependent, suggesting the involvement of different signaling pathways in different cell types (Lamouille et al., 2014). Although our data showed that tHGA can affect the phosphorylation of PI3K, it may be insufficient to prevent EMT in TGF-β-induced BEAS-2B.

To date, the mechanism implicated in eosinophil-epithelia contact interaction and the resulting EMT remains unknown. Models of eosinophil and epithelial cell co-culture have been utilized to elucidate the interaction between these cell types (Wang et al., 2005; Wong et al., 2013) with two studies demonstrating increased TGF-β production following co-culture (Mathur et al., 2013; Shimizu et al., 2014). However, upstream receptors/ligands that mediate cell-to-cell interaction/activation remain undefined. It has been proposed that the adhesion of eosinophils to airway epithelia is CD18/intercellular adhesion molecule 1 (ICAM-1) dependent (Burke-Gaffney and Hellewell, 1998). We have tested this hypothesis in the BEAS-2B/EoL-1 model through the use of a neutralizing antibody against ICAM-1 but failed to abrogate eosinophil-induced EMT (data not shown). Obviously further work is envisaged in this area. It is possible that tHGA may target further upstream molecules of JNK and PI3K. A better understanding of the mechanism orchestrating eosinophil-epithelial cell interaction may shed light on the exact molecular target of tHGA.

**Figure 8:**
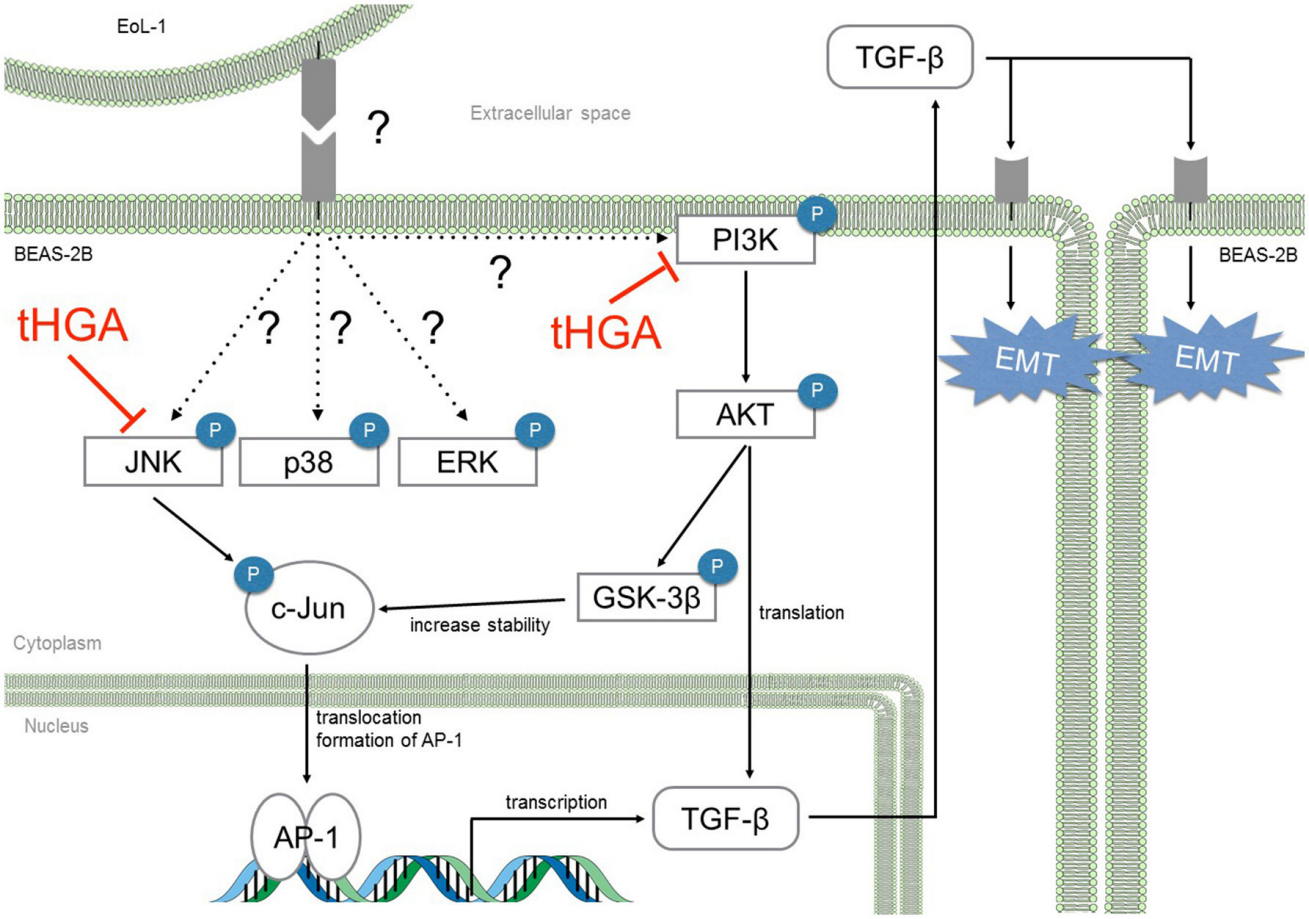
tHGA blocked eosinophil-induced EMT by suppressing TGF-β synthesis via JNK and PI3K pathway. tHGA's inhibition on JNK and PI3K phosphorylation affects downstream AKT, GSK-3β and c-Jun activation in bronchial epithelial cells. As a result, tHGA impedes the formation of active AP-1 that is involved in TGF-β gene transcription and subsequent synthesis. Suppression of AKT activation may also prevent TGF-β mRNA translation.

In conclusion, we demonstrated that tHGA blocked eosinophil-induced EMT by suppressing TGF-β synthesis via inhibition of JNK and PI3K phosphorylation and subsequent activation of AP-1 (Figure 8). This study strengthens our previous conclusions and provides further insights regarding the role of tHGA in attenuation of airway remodeling in experimental asthma (Lee et al., 2017).

## Author contributions

Participated in research design: YL, CT, MS, and DI. Conducted experiments: YL and HY. Contributed new reagents: KS, CT, MS, and DI. Performed data analysis: YL, HY, and DI. Wrote or contributed to the writing of the manuscript: YL, HY, KS, CT, MS, and DI.

### Conflict of interest statement

The authors declare that the research was conducted in the absence of any commercial or financial relationships that could be construed as a potential conflict of interest.
